# Frontier Breakthroughs: A Comprehensive Review of Diathermy in Dentistry With a Focus on Oral Medicine

**DOI:** 10.7759/cureus.57427

**Published:** 2024-04-01

**Authors:** Turaga Amani, Mouttoukichenin Surenthar, Roland Prethipa

**Affiliations:** 1 Oral Medicine and Radiology, Saveetha Dental College and Hospitals, Chennai, IND

**Keywords:** shortwave diathermy, musculoskeletal pain, oral medicine, myofacial pain, longwave diathermy, diathermy

## Abstract

Diathermy is a therapeutic technique utilizing electromagnetic waves that is widely used in the medical field, especially for orthopedic injuries such as musculoskeletal disorders. Shortwave diathermy (SWD), microwave diathermy (MWD), sonic therapy or ultrasound (US), and long-wave diathermy are the various types, out of which shortwave diathermy is most commonly used in medical fields. However, diathermy has not been explored much in dentistry. This literature review aims to discuss the various applications of diathermy and its potential use in dentistry with the existing scarce literature and further emphasize its role as a recommendation in the management of orofacial pain in dental practice.

## Introduction and background

Heat therapy is a widely used method that is divided into superficial and deep treatment. Superficial heat therapy, in which hot packs and thermal water are used, involves direct contact with the skin. In contrast, deep heat therapy is generated by the interaction of electromagnetic waves with biological tissue [1]. The US Food and Drug Administration (FDA) recognizes diathermy as a therapeutic method that produces profound heating under the skin, muscles, and joints for therapeutic applications [2]. Diathermy uses different types of electromagnetic waves such as high-frequency radio waves to produce heat within the body tissue.

Diathermy was developed in the late 19th century. Originally designed for heating deep tissue, it has evolved and is used in various medical fields [3]. This technique generates oscillating electromagnetic fields (EMFs), which are divided into microwave and shortwave frequencies. Despite its initial popularity, the use of diathermy has been limited in recent decades, possibly due to clinicians' hesitation and lack of knowledge of the relevant research [4].

In 1891, Nikola Tesla, an American engineer and inventor, first observed the generation of heat by irradiating tissue with high-frequency alternating current, particularly at wavelengths slightly longer than the longest radio waves. Tesla also pointed out the possible medical applications of this phenomenon. Later, in 1909, the German physician K.F. Nagelschmidt introduced the term "diathermy" to describe the process of "heating through" [5]. In the early 1930s, Esau and Schliephake in Germany and Shereshewsky in the USA pioneered ultrashort wave (USW) diathermy. This technique quickly became popular due to its effectiveness in selectively raising temperatures in deep tissue [6].

Applications of diathermy in various medical fields include physiotherapy, orthopedics, and rehabilitation. It is also used in specialist areas such as sports medicine and dermatology. In dentistry, it is used in orthodontics and oral and maxillofacial surgery. The technique's ability to heat tissue in depth and promote physiological reactions makes it versatile in the treatment of musculoskeletal, circulatory, and inflammatory disorders.

Orofacial pain is one of the most common conditions encountered in dental practice. Temporomandibular disorder (TMD) is a type of chronic orofacial pain condition that encompasses a range of disorders that cause discomfort in and around the temporomandibular joint (TMJ), limitations in jaw function, or clicking of the jaw joint during movement [7]. TMD commonly affects individuals between the ages of 20 and 40 and represents a group of clinical conditions affecting the TMJ, myofascial muscles, and associated tissues. The cause of TMD is multifactorial and includes biological, environmental, social, emotional, and cognitive reasons [8]. Although there are several traditional approaches for the treatment of orofacial pain, clinical practice guidelines are still a challenge [9].

This comprehensive review aims to shed light on the potential uses, principles, efficacy, and emerging trends of diathermy in the treatment of conditions related to orofacial pain, thereby filling a notable gap in the existing literature in the dental field. It provides a brief overview of the evidence supporting the clinical therapeutic use of diathermy as an oral medicine intervention for individuals with musculoskeletal disorders such as myofascial pain syndrome, myalgia, and myositis. To our knowledge, this review is a first in the scientific literature as it focuses on exploring the applications of diathermy in the field of dentistry, with particular emphasis on oral medicine.

## Review

Types of diathermy

The FDA categorizes diathermy into four types: shortwave diathermy (SWD), microwave diathermy (MWD), sonic therapy or ultrasound (US), and longwave diathermy [10].

Diathermy can be divided into four frequency categories: microwave, shortwave, sonic or ultrasound, and longwave. Shortwave diathermy is further subdivided into continuous (cSWD) or pulsed (pSWD) diathermy. In this method, oscillating electromagnetic fields (EMFs) are generated with electrical and magnetic components [11].

Continuous shortwave diathermy (cSWD) is primarily used for its thermal effects, which lead to vasodilation, an increased pain threshold, a reduction in muscle spasms, accelerated cell metabolism, and improved extensibility of soft tissue [12]. In contrast, pulsed shortwave diathermy (pSWD) is used for non-thermal effects, where the athermal effects are likely due to the cells absorbing the energy of the oscillating electric fields, thereby inducing or intensifying cellular activity. These effects include increased blood flow, relief of joint pain and stiffness, reduction of inflammation, faster resolution of edema, and improved wound healing [13].

Shortwave diathermy (SWD) generates intense heat in the subcutaneous tissue through oscillating radio frequency electromagnetic fields, typically at 13.56 or 27.12 MHz, using two capacitor probes [6]. Microwave diathermy (MWD), which works with electromagnetic waves in the 915-2456 MHz range, stimulates molecules in the target tissue and converts electrical energy into heat. MWD is particularly effective in tissues with high water content and is usually administered with a single emitter. Capacitive-resistive electric transfer (CRet) therapy is used to treat injuries to the musculoskeletal system. It can be categorized as longwave diathermy and is a noninvasive electrothermal treatment in which electric currents in the radio frequency range of 300 kHz-1.2 MHz are applied [10]. Ultrasound therapy, which falls under the category of diathermy, is sometimes combined with electrical stimulation. For effective ultrasound diathermy, it is necessary to work at a frequency between 800 and 1,000 kHz [14].

Principle of diathermy

**Figure 1:**
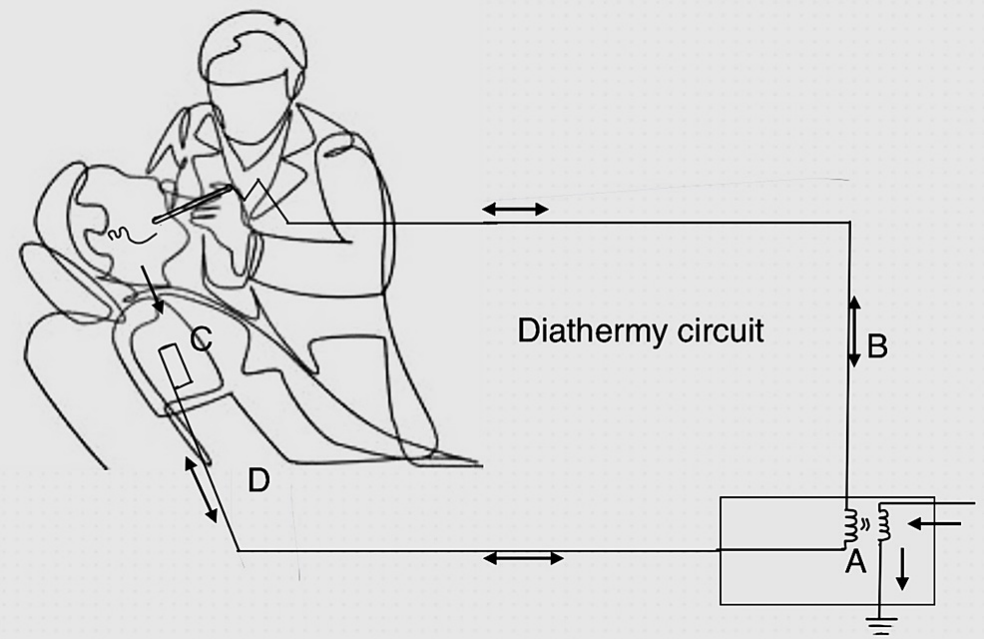
Diathermy circuit A: generator, B: active electrode, C: body, D: passive dispersive electrode Picture courtesy: Amani T

Electric current flows through the body via electrolytic solutions in the tissues and bloodstream. The diathermy generator produces the source voltage by converting the 50 Hz main current into a high-frequency current in the range of 0.2-3 MHz. At these radio frequencies, the risk of muscle or nerve stimulation is minimal, as the electrical energy is mainly converted into heat when the electrons overcome the body impedance. The current density, i.e., the current per unit cross-section, influences the effect. It is more pronounced when it is concentrated at a single point, such as the active electrode (surgical tool), than when it is spread over a large area, such as the dispersive electrode plate. The tissue temperature at the active electrode tip reaches about 1000°C, while at a distance of 1 cm, it only reaches about 38°C. Despite a constant current at a given voltage, a reduced area for current flow increases resistance, resulting in increased heat production [14]. Figure 1 shows a diathermy circuit [15].

**Figure 2:**
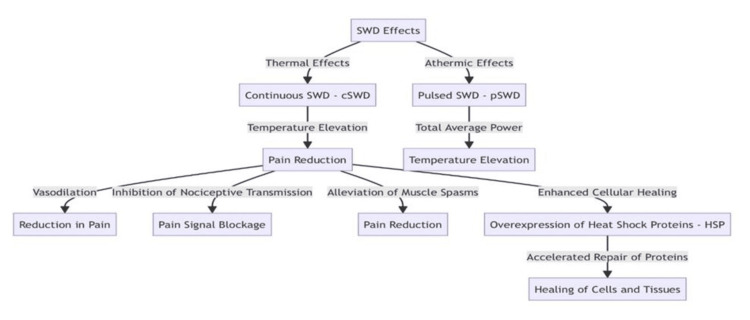
Flowchart explaining the principle of shortwave diathermy SWD: shortwave diathermy, cSWD: continuous shortwave diathermy, pSWD: pulsed shortwave diathermy, HSPs: heat shock proteins Flowchart courtesy: Amani T, Surenthar M, Prethipa R

Continuous shortwave diathermy (cSWD) primarily causes thermal effects, while pulsed shortwave diathermy (pSWD) is associated with athermal effects. Recent studies show that pSWD can lead to an increase in tissue temperature, which depends on the total average power emitted [12]. Despite debates about the actual impact of athermal phenomena, the clinical effects of SWD are often associated with an increase in temperature. Pain relief, an important outcome of diathermy, is not fully understood physiologically. Heat-induced pain relief may be due to vasodilation, which facilitates the outflow of pain mediators such as bradykinin, serotonin, and prostaglandins. Another possible mechanism is the inhibition of nociceptive transmission by the gate control system, which blocks pain signaling in the spinal cord [16]. In addition, SWD's ability to relieve muscle spasms contributes to pain relief and promotes cellular healing processes by promoting the overexpression of heat shock proteins (HSPs). Increased HSP levels accelerate the repair of intracellular proteins and thus accelerate the healing of cells and tissues [17]. Figure 2 describes the principle of shortwave diathermy in the form of a flowchart.

Applications in other fields

Despite its most common use in medical care, diathermy is also used in various other fields of science and industry. In telecommunications, diathermy has been historically used for radio frequency heating and in certain industrial processes. In material science, it is employed for specific heat treatments and metallurgical applications. The technology's ability to induce controlled heat in targeted areas continues to have diverse applications beyond the medical sector, demonstrating its versatility in a variety of scientific contexts and industries [18].

Applications in the medical field

Diathermy is used in musculoskeletal disorders such as osteoarthritis, tendonitis, tenosynovitis, joint contractures, decreased joint stiffness, myofascial trigger points, cervical spondylosis, plantar fasciitis, and subacute and chronic pain [1,4,14]. Surgical diathermy is used for cutting, coagulation, fulguration, desiccation, blend-in eye surgery, neurosurgery, and dermatology [15].

Applications in dentistry

Limited literature is available on the usage of diathermy in the dental field. Until now, microwave diathermy was used in orthodontics in a case report by Cirulli et al., a single-pole resistive session at 1,000 kHz, set to 7% power in "fracture consolidation treatment" mode, which was conducted for six minutes. This diathermy stimulation occurred weekly on the same side over three weeks. By the end of the fourth week, positive outcomes in translation, angulation, rotation, and torque were observed facilitating tooth movement. Based on this study's findings, diathermy may be a useful tool for accelerating orthodontic movement, although its effects would be minimal compared to the control side [19]. According to Royer et al., microwave diathermy was applied for 20 minutes at 100 watts, 5 cm from the cheek, and was given on one side for the first four days post-surgery; it has shown a reduction in postoperative swelling and trismus in patients who had undergone odontectomy of third molars [20]. In a study by Wahab et al., an incision in patients who underwent Le Fort I or anterior maxillary osteotomy was given by diathermy, and there was minimal blood loss when compared to a scalpel incision [21]. According to Kalekar et al., a continuous mode of shortwave diathermy was given for a total duration of 30 minutes per session for five consecutive days for patients with chronic sinusitis and showed significant symptomatic relief [22].

There were fewer studies where shortwave diathermy was used for TMJ disorders. Gray et al. used shortwave diathermy in a mild thermal setting that was applied for 10 minutes for TMD patients, and it showed significant relief of pain in those patients [23]. In a study by Dhanasekaran et al., pulsed shortwave therapy (pSWT) operated at 27.12 MHz, delivered short pulses of a magnetic field at a frequency of 1,000 pulses per second for five consecutive days, and showed good pain relief without adverse effects in TMD patients [24]. A case report by Tuli et al. reported the use of monopolar diathermy to release the frenum in a patient with ankyloglossia, which resulted in good postoperative hemostasis [25]. Current literature has shown the use of diathermy in various musculoskeletal disorders such as osteoarthritis, shoulder tendinopathies, lower back pain, lower limb tendinopathies, and neck pain, which resulted in good pain reduction. From this evidence, diathermy can potentially be considered a therapeutic option for patients with orofacial pain caused by musculoskeletal disorders such as arthralgia, internal derangement of the TMJ, myalgia, myofascial pain, muscular contracture, and muscle hypertrophy by its thermal and athermic effects.

Recommendations of diathermy in the oral medicine field

Diathermy could be a recommended treatment for orofacial pain based on the limited evidence found in the literature. The current approach to the treatment of orofacial pain consists primarily of systemic medications such as nonsteroidal anti-inflammatory drugs (NSAIDs) and muscle relaxants [26]. Diathermy is a noninvasive therapeutic method that can provide relief from orofacial pain by promoting vasodilation and improving blood flow to the affected area. This improved circulation can contribute to pain relief. The heat generated by diathermy has a muscle-relaxing effect by promoting vasodilation, inhibiting nociceptive transmission, alleviating spasms, and enhancing the cellular healing mechanism, which thereby reduces muscle spasms and tension. This may be beneficial for individuals suffering from orofacial pain due to muscle tension or dysfunction and may speed the resolution of certain inflammatory conditions or injuries that contribute to orofacial pain. Diathermy can be used as an intervention in the treatment of orofacial pain due to its potentially faster onset of action, good patient tolerance, and reduced systemic side effects. For patients with temporomandibular disorders, shortwave diathermy at 27.12 MHz in continuous or pulsed mode is recommended three times a week for 15-20 minutes over a period of 2-3 weeks. The treatment's outcomes include pre- and post-treatment visual analog scale (VAS) evaluations, as well as a follow-up at one, three, and six months after treatment [1,2]. Treatment using shortwave and longwave diathermy is not particularly costly. Typically, in India, the procedure will cost between Rs. 5,000 and Rs. 30,000.

Disadvantages of diathermy

The administration of uncontrolled heat leads to tissue damage. Consequently, not only are the intended target tissues exposed to the heat, but the surrounding tissues are also affected, leading to undesirable consequences. The subcutaneous layer of the skin is rich in fatty tissue, which makes it difficult to conduct heat directly into deeper tissue layers. The electromagnetic (EM) energy used in diathermy can generate excessive heat in metal devices such as bone nails, dental fillings, and metal sutures, which can lead to burns, electrocution, hypoxic stress, inhalation of diathermy plumes, and genetic mutations [27,28].

Contraindications of diathermy

Diathermy is contraindicated if artificial implants such as metal implants, pacemakers, and intrauterine devices are present. It is also not recommended for cardiovascular conditions, including ischemic tissue, hemorrhage, and venous thrombosis, and conditions such as synovitis, acute injuries, osteomyelitis, tuberculosis, malignancy, and pregnancy. The majority of dental procedures are safe, and there have never been any documented side effects from using over dental implants, metal bridgeworks, and other dental fillings [29].

Future studies should focus on the use of diathermy as an intervention for patients with orofacial pain to know the efficacy and effectiveness of diathermy as a therapeutic modality in the management of orofacial pain.

## Conclusions

Based on the current literature review, diathermy, a therapeutic technique involving the application of heat through high-frequency electric currents, may potentially be used as a viable option for the treatment of orofacial pain. However, it is important to note that diathermy should only be used with the necessary precautions. To further evaluate the efficacy of diathermy, large-scale randomized controlled trials using diathermy as an intervention are needed. These studies would serve to evaluate the efficacy of diathermy in the treatment of orofacial pain conditions such as arthralgia, temporomandibular joint (TMJ) internal derangements, myositis, myalgia, and myofascial dysfunction.
